# Mortality in Rheumatoid Arthritis (RA): factors associated with recording RA on death certificates

**DOI:** 10.1186/s12891-015-0727-7

**Published:** 2015-10-05

**Authors:** Emily Molina, Inmaculada del Rincon, Jose Felix Restrepo, Daniel F. Battafarano, Agustin Escalante

**Affiliations:** University of Texas Health Science Center at San Antonio, 7703 Floyd Curl Drive, San Antonio, TX 78229 USA; San Antonio Military Medical Center, San Antonio, TX USA

**Keywords:** Rheumatoid arthritis, Death certificates, Mortality, Epidemiology

## Abstract

**Background:**

Death certificates can be used to assess disease prevalence and incidence; however, rheumatoid arthritis (RA) often remains unreported in death certificates. We sought to determine to what extent RA is underreported and what demographic and clinical characteristics could predict mention of RA in the death certificate.

**Methods:**

We recruited 1328 patients with RA from private, public and military rheumatology practices and followed them prospectively for yearly evaluations. A rheumatologist assessed clinical characteristics of RA and comorbidities at each evaluation. Deaths were identified through family members, other physicians, obituaries and public death databases. All were confirmed with state-issued death certificates. Patients with and without RA in death certificate were compared using bivariate and multivariate analyses.

**Results:**

By December 2013, 326 deaths had occurred. We received and reviewed death certificates for all confirmed deaths, of which 58 (17.7 %) mentioned RA on the death certificate. Bivariate analysis revealed that younger age, a greater number of deformities, higher Sharp score and lower socioeconomic status were each associated with recording RA. Multivariable analyses revealed that comorbidity [OR (95 % CI) = 0.84 (0.73, 0.97); *P* = 0.022] was inversely associated with listing RA on the death certificate, while the number of deformities [OR (95 % CI) = 1.04 (1.00, 1.07); *P* = 0.033] and a certified physician’s signature on the death certificate [OR (95 % CI) = 4.79 (1.35, 16.9); *P* = 0.015] increased likelihood of reporting RA.

**Conclusion:**

In this cohort, RA was not listed in over 80 % of death certificates. Younger patients with fewer comorbidities and more joint deformities were more likely to have RA reported.

**Discussion:**

RA is often not included in death certificates. The findings of this study suggest that older patients may have a greater number of comorbidities, thus decreasing the likelihood that RA be included when completing the death certificate.

## Background

Rheumatoid arthritis (RA) is a chronic disease characterized by joint inflammation that affects approximately 1 % of Americans [1]. Although ultimate causes of death in RA patients are similar to the general population [2], mortality rates in RA patients are 1.5 – 1.6 times higher [2, 3]. However, despite such a high mortality, previous studies have indicated that RA may be underreported in death certificates with anywhere between 50 and 89 % lacking mention of the disease [2, 4–6]. The tendency to under-report RA on death certificates has persisted since it was first reported half a century ago [2].

Death certificates are important tools used in epidemiological research. Yet physicians completing them may be unaware of underlying conditions. Although rheumatic diseases such as RA are not often considered immediate causes of death, they may predispose patients to comorbidity that may directly cause mortality, such as cardiovascular (CV) disease [7, 8]. Moreover, some commonly used treatments may predispose RA patients to burdensome side effects [9, 10] and increased mortality [11]. Without identifying RA as an underlying condition, epidemiological studies that rely on information from death certificates may not fully ascertain the morbidity and mortality of the disease.

In this study, we examine frequency of reporting RA and what factors may predict recording of RA on the death certificate.

## Methods

### Patients

From 1996 to 2009, we recruited consecutive patients who met the 1987 criteria for RA [12] from private, public and military rheumatology practices in San Antonio, Texas. We have described part of this cohort in previous publications [13–15]. All patients granted informed consent before participating in a comprehensive baseline evaluation of their clinical and psychosocial characteristics conducted by a physician and trained research assistants. Afterwards, we invited them for annual follow-up evaluations through the censor date of December 31, 2013. Throughout 2014, we underwent further efforts to ascertain vital status of patients by contacting patients and families directly. We learned about deaths from public databases, physicians, relatives, neighbors and obituaries. All deaths were confirmed by death certificate. This study received approval from the institutional review board at the University of Texas Health Science Center at San Antonio.

### Demographics

A trained interviewer asked patients for their date and place of birth, sex and race/ethnicity. For the latter, the interviewer asked patients to self-identify as “white,” “black,” “Asian,” “Hispanic,” or “other.”

### Socioeconomic status

We classified socioeconomic status (SES) according to Nam and Powers, using years of formal education, inflation-adjusted monthly household income, and current or past occupation at baseline to calculate an SES score on an ascending scale between 0 and 100 [16]. Health insurance information was obtained at each evaluation. Analyses included insurance information from the most recent visit prior to death.

### Clinical features

#### Body Mass Index (BMI)

Obesity was measured at each encounter by assessing BMI, which is calculated as weight in kilograms divided by the square of the height in meters. In this study, we used the BMI from the most recent visit.

#### Joint findings

A physician examined 48 joints for tenderness or pain on motion, swelling or deformity, and for the presence of extra-articular subcutaneous nodules [17]. Joint exam Spearman-Brown reliability coefficients are 0.94 for tender/pain on motion, 0.90 for swelling and 0.98 for deformity [18]. In these analyses, we used the 28 joints included in the DAS28ESR from the most recent visit.

#### Hand radiographs

We quantified joint damage on a plain X-ray view of both hands and wrists according to Sharp and colleagues [19]. In this study, all X-ray radiographs were scored by one rater (JFR). Analyses included radiographs from the most recent visit prior to death.

#### Rheumatoid nodules

Absence or presence of rheumatoid nodules was determined by a rheumatologist at each patient evaluation. In this study, nodules were considered present if they were found in the most recent visit prior to death.

#### Steinbrocker functional class

The Steinbrocker functional classification [20] was assigned by the physician or a research nurse who was trained in physical function assessment. The Steinbrocker functional classification is used to rate the extent of physical disability on a 4-level scale. Spearman-Brown reliability of the Steinbrocker classification in our study was 0.84.

#### Comorbidity

The comorbidity scale that we used was the Charlson Comorbidity Index [21]. This index records the presence or absence of 18 health problems, each one weighted for severity according to predefined values. The final score is provided by the sum of the weights of each patient’s health problems. The examining physician used a validated self-report questionnaire to interview the patients about the presence or absence of the predefined health problems [22]. The physician then reviewed available medical records to verify the problems reported by the patient. If discrepancies between the medical record and the self-reported responses arose, the case was discussed among 3 or 4 physicians in order to decide on the final score. Interrater reliability was 0.94 [23].

### Information on the death certificate

To confirm deaths, state-issued death certificates were obtained from the state in which the patient died. Immediate cause of death listed and any underlying conditions were coded using the International Classification of Diseases, Version 9 (ICD-9) by an experienced nosologist. We recorded whether a certified physician, medical examiner or a justice of the peace signed the death certificate. Place of death was ascertained by the death certificate and was categorized as either “home,” “hospital,” “nursing home,” or “other.” We further noted if an autopsy was performed and if those results were available at the time the death certificate was written.

### Statistical analysis

Bivariate analyses were conducted to compare patients with and without RA recorded on the death certificate. We assessed differences in demographics, socioeconomic status, and clinical characteristics between the two groups using t-tests. We further conducted a series of multivariable logistic regressions to identify which factors are independently associated with recording RA. In the final multivariable model, we included variables that were found to be significant in bivariate analyses as well as potential confounders (sex, ethnicity, socioeconomic status, etc.). Moreover, because the Charlson Comorbidity score is not linearly distributed, we divided the variable into tertiles for the final multivariable model. When examining overall mortality in RA, we calculated death rate for all-cause and CV mortality.

All analyses were conducted using a desktop personal computer with the Stata statistical software package, version 9.0 (College Station, TX).

## Results

There were a total of 1328 participants recruited between 1996 and 2009. By December 2013, 326 deaths had occurred during 8966 person-years of observation, for a mortality rate of 3.6 per 100 person-years [95 % confidence interval (CI) (3.3, 4.0)]. Average age (range) at death was 71.8 years (46.1 - 92.8 years). Moreover, most of the patients (55.5 %) died in a hospital, though there were no significant differences in immediate cause of death by the place of death. Of the 326 death certificates we received, 58 (17.7 %) mentioned RA on the death certificate. Of the 58 certificates, 2 recorded RA as the immediate cause of death, 22 recorded it as a cause leading up to death, and 34 recorded it as an underlying condition not directly contributing to the cause of death.

Because we followed patients for numerous visits over several years, we used the most recent information prior to death. Average time between death and the most recent evaluation was 2.19 years (0.01 – 10.1 years). By comparing patients with and without RA listed on the death certificate, we found that patients who were younger at the time of death (*P*-value = 0.05), had lower socioeconomic status (*P* = 0.011), a greater number of deformities (*P* = 0.008), and higher Sharp score (*P* = 0.003) were more likely to have RA recorded on the death certificate (Table 1). There were no significant differences in sex, ethnicity or presence of health insurance between the two groups. Other factors such as immediate cause of death, place of death, presence of rheumatoid nodules, and performing an autopsy were also not associated with recording RA in bivariate analyses.

**Table 1:** Bivariate analyses of 326 RA patients with and without RA on the death certificate

	RA recorded		
Demographics	Yes (*n* = 58)	No (*n* = 268)	*P*-value
Age in years at time of death, mean (SD)	69.3 (9.8)	72.3 (10.1)	0.05
Women, n(%)	36 (62.1)	161 (60.3)	.8
Ethnicity, n (%)			
White	23 (39.7)	127 (47.6)	.3
Black	2 (3.4)	16 (6.0)	-
Asian	2 (3.4)	1 (0.4)	-
Hispanic	31 (53.4)	123 (45.9)	0.3
Other	0	1 (0.4)	-
Socioeconomic status			
Nam & Powers Score, mean (SD)	41.0 (2.8)	48.4 (1.20)	0.011
Had insurance at most recent visit, n(%)	54 (93.1)	259 (96.6)	.2
Clinical characteristics			
Disease duration in years, mean (SD)	22.7 (13.0)	21.6 (12.0)	0.5
Presence of rheumatoid nodules, n(%)	37 (63.7)	148 (55.2)	0.2
Deformities of 28 joints, mean (SD)	17.6 (9.3)	14.0 (9.2)	0.007
Sharp Score^a^, mean (SD)	196.2 (133)	135.6 (109)	0.003
Steinbrocker functional class			
Class II, n(%)	19 (32.8)	121 (45.1)	0.08
Class III, n(%)	24 (41.4)	95 (35.6)	0.4
Class IV, n(%)	14 (24.1)	40 (15.0)	0.08
Charlson Comorbidity Index, mean (SD)	4.09 (2.3)	4.57 (2.4)	0.2
BMI, mean (SD)	26.0 (6.9)	27.5 (6.4)	0.1
Death certificates			
Autopsy Performed, n (%)	3 (5.1)	16 (5.9)	0.8
Who signed Death Certificate, n(%)			
Certified Physician	55 (94.8)	228 (85.1)	0.046
Medical Examiner	3 (5.2)	36 (9.8)	0.08
Justice of the Peace	0 (0)	4 (1.5)	0.4
Place of Death, n (%)			
Home	17 (29.3)	70 (26.1)	0.6
Hospital	33 (56.9)	148 (55.4)	0.8
Nursing Home	5 (8.6)	30 (11.2)	0.6
Other	3 (5.2)	20 (7.5)	0.5
Primary Cause of Death, n(%)			
Cardiovascular (*n* = 111)	15 (25.9)	96 (35.8)	0.1
Respiratory (*n* = 68)	16 (27.6)	52 (19.5)	0.2
Neoplasms (*n* = 51)	12 (20.7)	39 (14.6)	0.2
Infection (*n* = 27)	5 (8.6)	22 (8.2)	0.9
Rheumatoid Arthritis (*n* = 2)	2 (3.4)	0	-
Other (*n* = 67)	8 (13.8)	59 (22.1)	0.2

^a^Hand radiographs were not available for 76 of the 326 deceased RA patients. Of these, 20 had RA listed on the death certificate, while 56 did not

We sought to determine what factors were independently associated with recording RA through multivariable analyses. Because we found that having a greater number of deformities [OR (95 % CI) = 1.04 per deformity (1.00, 1.07); *P* = 0.033] and a fewer number of comorbidities [OR (95 % CI) = 0.84 (0.73, 0.97); *P* = 0.022] increased likelihood of reporting RA after adjusting for confounders such as age, sex, ethnicity and socioeconomic status, in our final model, we divided both of these variables into tertiles (Table 2). At the time of death, 112 patients had between 0 and 9 deformities, 108 patients had between 10 and 21, and 106 patients had between 22 and 28. Patients in the tertile with the greatest number of deformities were 2.48 times more likely to have RA recorded than those in the tertile with the fewest deformities (Table 2). There were 127 patients with a Charlson comorbidity score between 1 and 3, 101 between 4 and 5, and 98 between 6 and 14. In this final model, we found that age [OR (95 % CI) = 0.97 (0.94, 1.00); *P* = 0.06] no longer remained significantly inversely associated with having RA recorded. However, compared to patients with the least comorbidity, those with the most were significantly less likely to have RA mentioned on the death certificate [OR (95 % CI) = 0.32 (0.14, 0.75); *P* = 0.008]. Moreover, in this model, having a certified physician sign the certificate [OR (95 % CI) = 4.97 (1.39, 17.7); *P* = 0.013] increased the likelihood that RA would be reported (Table 2).

**Table 2:** Multivariable analyses of factors associated with recording RA in death certificates (*n* = 326)

Independent variable	Odds ratio (OR)	95 % CI	*P-*value
Age	0.97	0.94, 1.00	0.06
Sex (Female==1)	0.78	0.41, 1.51	0.5
Non-Hispanic White ethnicity	1.1	0.56, 2.19	0.8
Health Insurance (yes==1, no==0)	0.57	0.15, 2.10	0.4
Nam & Powers Score	0.98	0.97, 1.00	0.1
Number of Deformities (0–9)	1.0	REFERENT	–
Number of Deformities (10–21)	1.01	0.45, 2.33	0.9
Number of Deformities (22–28)	2.48	1.1, 5.6	0.03
Steinbrocker Functional Class	1.0	REFERENT	–
Class II	1.53	0.18, 13.4	0.7
Class III	2.13	0.23, 19.4	0.5
Class IV	1.90	0.19, 18.7	0.6
Charlson Comorbidity (range 1–3)	1.0	REFERENT	–
Charlson Comorbidity (range 4–5)	0.82	0.40, 1.68	0.6
Charlson Comorbidity (range 6–14)	0.32	0.14, 0.75	0.008
Signed by Certified Physician	4.97	1.39, 17.7	0.013

The most common immediate cause of death was CV-related, with 111 (34.0 %) deaths resulting from disease of the circulatory system (ICD-9 codes between 390 and 459) (Table 1). However, we found an additional 61 death certificates listed CV disease as an underlying condition, summing to a total of 171 (52.6 %) of deaths resulting from CV causes (Table 3). Interestingly, although only 58 death certificates listed RA, an additional 10 death certificates mentioned other musculoskeletal diseases (ICD-9 codes between 710 and 739) such as systemic lupus erythematosus, CREST (calcinosis, Raynaud phenomenon, esophageal dysmotility, sclerodactyly, and telangiectasia) syndrome or mixed connective tissue disease without mentioning RA (Table 3), likely representing overlap conditions with RA. None of these 10 patients received autopsies. We reviewed our comorbidity data and confirmed that these patients had overlapping musculoskeletal conditions.

**Table 3:** Immediate cause of death and underlying conditions found in 326 RA death certificates by ICD-9 code

Condition (ICD-9 code range)	Total death certificates listing condition (%)^a^
Infectious & Parasitic Diseases (001 – 139)	55 (16.9 %)
Neoplasms (140 – 239)	69 (21.2 %)
Diseases of the Circulatory System (390 – 459)	172 (52.7 %)
Diseases of the Respiratory System (460 – 519)	123 (37.8 %)
Diseases of the Musculoskeletal System^b^ (710 – 739)	68 (20.9 %)

^a^Percentages add up to more than 100 % as many patients had more than one underlying condition across several ICD-9 code categories

^b^Ten patients have ICD-9 codes for Diseases of the Musculoskeletal System listed on the death certificate, but do not list RA

## Discussion

Previous studies have suggested that death certificates in RA may be unreliable [2, 4–6]. This was first noted in 1967, by Atwater and Jacox, who found that less than 50 % of death certificates for RA patients mentioned the disease [5]. Since then, several studies have found similar results [2, 4, 6]. In 2008, Sokka et al. reviewed over 50 cohorts between the years 1953–2008 and found that little has changed in the past 30 years, with only an average of 9.4 % death certificates in RA mentioning musculoskeletal disease [2]. In our study, we evaluated 326 death certificates and not only confirmed that RA remains widely under-reported, but also assessed what predictive factors may contribute to successfully indicating the presence of RA in vital statistics. One unique aspect of our study is the availability of detailed clinical and laboratory ante-mortem data. This information allowed us to examine factors associated with reporting of RA.

We describe demographic and clinical characteristics of 326 RA patients and their corresponding death certificates. On average, patients with RA recorded in the death certificate were younger at death, had a greater number of joint deformities, and a higher Sharp score (Table 1). These results are not surprising because RA becomes more obvious to the physician or medical examiner when patients have a greater number of deformities. Patients with fewer deformities may not be as readily identified as arthritis patients.

Moreover, patients who were younger at death may have less comorbidity to report, thus bringing chronic rheumatic diseases to the forefront of relevant diagnoses to mention in the death certificate. A study examining the reporting of systemic lupus erythematosus (SLE) mortality noted that 40 % of death certificates lacked mention of SLE [24]. These authors found that older patients and those without health insurance were less likely to have SLE recorded. Although in our RA cohort there was no indication that presence of health insurance affected the completeness of the death certificate in regards to RA, we did find similar results regarding age (Table 1). However, our final analytical model suggests lower comorbidity in RA is the underlying reason for this association, as a lower comorbidity score remains independently associated with recording RA, not older age (Table 2). Older patients may have a greater number of comorbidities, thus increasing the likelihood that RA could be overlooked when completing the death certificate. As the average age of RA onset increases [1], this may bias future studies on mortality in RA towards younger patients with more severe disease and less comorbidity.

Furthermore, aside from greater joint deformities and less comorbidity, the only other variable analyzed that independently correlated with recording RA on the death certificate was having a certified physician signed the death certificate (Table 2). Of the 58 death certificates that reported RA, a certified physician signed 55. At first, this result was surprising; medical examiners require a medical degree and therefore are well versed in medicine as well as forensic science. However, when a certified physician signs, they are more likely to know the patient history compared to a medical examiner or justice of the peace [25]. Therefore, regardless of the number of discernible joint deformities, these physicians are familiar with a patient’s case and can consequently report RA accordingly. Nevertheless, while having a certified physician sign increased likelihood of RA being reported in our cohort, still only 55 (19.5 %) of the 282 death certificates signed by a certified physician listed RA at all.

Although death certificates were not originally intended for epidemiological research, they have become useful tools when examining mortality in population and cohort studies. Therefore, incomplete or inaccurate records have many implications for research that rely on these records. Given that diseases of the musculoskeletal system are not often considered immediate causes of death, completeness of recording underlying conditions in the death certificate becomes crucial when analyzing mortality of these diseases. Although many patients may not die directly from RA and therefore do not have it included as an immediate cause of death or as a cause leading up to death, there is a separate section for underlying chronic conditions where RA may still be mentioned.

A number of studies have suggested that the higher mortality in RA compared to the general population has not decreased over the past several decades [3, 26], with CV disease being the most common cause of death [2]. Our results further suggest CV disease remains the most common cause of death with 52.6 % of death certificates listing a disease of the circulatory system (ICD-9 codes 390 – 459) as the immediate or underlying cause of death (Table 3). Nevertheless, it has been noted in the general population that CV disease may be over-reported in death certificates [27].

Interestingly, although only 58 death certificates listed RA in the total cohort, an additional 10 listed other musculoskeletal conditions. Although none of these 10 patients received autopsies, certified physicians signed all of these death certificates. However, when reviewing comorbidity in each of these cases, all 10 patients had overlap with another musculoskeletal disease, such as lupus, mixed connective tissue disease or Sjögren’s syndrome. The overlap in these diseases is thus an additional barrier to completing the death certificate with accurate representation of RA.

Our study on the reporting of death certificates has several strengths and weaknesses. We have mentioned the rich variety of ante-mortem data that allowed us to examine the patient characteristics associated with RA reporting. Moreover, we recruited participants from several public, private and military health clinics throughout San Antonio, resulting in a diverse cohort from a variety of backgrounds. Because our patients were all recruited from San Antonio, Texas, the geographic coverage may limit our generalizability. However, underreporting of rheumatic diseases in death certificates has been noted from patients recruited in other states [5, 24], as well as other countries [4, 6].

## Conclusions

The present study suggests that RA is often underreported in death certificates, with less than 20 % of death certificates from our cohort accurately mentioning the disease. Studies of mortality in RA that rely on death certificates may be biased towards younger patients with more severe RA and less comorbidity. More research is needed to determine what other factors may explain this underrepresentation, as well as what may prevent this discrepancy in the future.

## Abbreviations

BMI: Body mass index
CI: Confidence interval
CREST: Calcinosis, Raynaud phenomenon, esophageal dysmotility, sclerodactyly, and telangiectasia
CV: Cardiovascular
ICD-9: International Classification of Diseases, 9th Revision
OR: Odds ratio
RA: Rheumatoid arthritis
SES: Socioeconomic status
SLE: Systemic lupus erythematosus
